# Fine-scale conditions across mangrove microhabitats and larval ontogeny contributes to the thermal physiology of early stage brachyurans (Crustacea: Decapoda)

**DOI:** 10.1093/conphys/coab010

**Published:** 2021-03-16

**Authors:** L D Vorsatz, P Pattrick, F Porri

**Affiliations:** 1 Department of Zoology and Entomology, Rhodes University, Makhanda 6140, South Africa; 2 South African Institute for Aquatic Biodiversity (SAIAB), Makhanda 6139, South Africa; 3 The Swire Institute of Marine Science and the Division of Ecology and Biodiversity, The University of Hong Kong, Pokfulam Road, Hong Kong, Hong Kong SAR; 4 South African Environmental Observation Network, Elwandle Coastal Node, Port Elizabeth 6070, South Africa

**Keywords:** Development, habitat complexity, larvae, mangroves, respirometry, vulnerability

## Abstract

Most marine ectotherms require the successful completion of a biphasic larval stage to recruit into adult populations. Recruitment of larvae into benthic habitats largely depends on biological interactions and favourable environmental conditions such as the inescapable diurnal thermal and tidal exposures. Hence, assessing how different taxa metabolically respond to variations in temperature is imperative to understand the community and ecosystem dynamics at both local and global scales. The present study aimed to investigate the effects of acute temperature variation on the physiology of stage-specific brachyuran larvae collected from different microhabitats at two mangrove forests in South Africa. Results indicate that the conditions within microhabitats, which larvae experience, likely influence their physiology, based on respirometry, to short-term acute temperature exposures. Furthermore, the larval thermal optimum shifted ontogenetically to become increasingly eurythermic as individuals developed from stage I zoea through to megalopa. Mangrove crab larvae in their early stages are hence increasingly vulnerable to acute temperature exposures, which could be particularly harmful to the persistence of populations if thermally stressful events increase in magnitude and frequency.

## Teaser

Small-scale variations in microhabitat conditions influence the physiology of mangrove-associated crab larvae throughout their ontogeny. The zoeal stage is the most vulnerable as a bottleneck for successful keystone species’ larval development in subtropical estuaries if climate anomalies persist in an already harsh intertidal environment.

## Introduction

Temperature affects all levels of biological organization and closely influences the physiology of aquatic ectotherms (Angilletta, 2009, Donelson et al. 2019, Harada and Burton 2019). Thus, the ability of an organism to adapt to rapid variations in temperature possibly plays a more significant role in climate change adaptation than its response to average rising thermal stress (Clusella-Trullas *et al.*, 2011; Harada and Burton, 2019; Scheffler *et al.*, 2019). Variations in water temperature are inescapable for aquatic ectotherms; this is exacerbated within intertidal habitats that experience diurnal fluctuations in both temperature and water level. Assessing how aquatic ectotherms metabolically respond to short- and long-term variations in temperature is therefore crucial to understand the community and ecosystem dynamics at both local and global scales (Walther *et al.*, 2002; Harley *et al.*, 2006).

The majority of macrobenthic marine, coastal and estuarine invertebrate groups have complex life cycles and possess a reproductive strategy that produces dispersing meroplanktonic larvae (Anger, 2006). Larval stages of macrobenthic invertebrates are morphologically and ecologically distinct from adults and undergo successive developmental stages through metamorphosis (for a review, see Pechenik, 1999). The dispersing meroplanktonic larvae play a significant role in determining the potential and extent of benthic habitat expansion or recolonization, contribute to the stabilization of food webs by being part of the diet of planktonic predators and stabilize benthic adult populations by reducing the variability in population density (Anger, 2006; Shanks, 2009; Saintilan and Mazumder, 2017). Environmental filtering shapes the spatio-temporal distribution of larval populations and communities, resulting in wide-scale patterns of larvae concentrated in areas with optimal conditions that are favourable to their development (Epifanio and Cohen, 2016). Temperature, along with salinity, is the most influential physico-chemical factors in larval survival and development (Magris and Fernandes, 2011). It is thus expected, that physiological temperature constraints, along with other environmental and biotic factors, shape the fitness of populations through either enhancing or impairing the metabolic rates of benthic invertebrate larvae (Brown *et al.*, 2004).

Brachyurans have complex life history strategies usually comprising of zoeal and megalopal stages, of which both need to be completed successfully for recruitment into adult populations (Byrne, 2012). The persistence of brachyuran populations hence relies on such a supply of planktonic larvae (Miller and Shanks, 2004). These life history traits and subsequent population dynamics of brachyuran crabs are thus directly expressed through physiological processes and can therefore be linked to the environment experienced by the individuals (Young *et al.*, 2006; Small *et al.*, 2015). The environment experienced in early life stages can potentially impose irreversible changes in individual phenotypes and long-term fitness (Burton and Metcalfe, 2014). The latent effects of an organism’s environmental history can therefore be observed in the adjustment of temperature and salinity tolerance of individuals from habitats with variable food concentrations (productivity), dissolved oxygen, flow, turbulence, etc. (for a review, see Anger, 2006). The thermal physiology of brachyuran larvae is particularly interesting given that life stages may differ in their vulnerability to environmental challenges (Kroeker *et al.*, 2013). Understanding the physiological responses to acute temperatures changes, throughout larval ontogeny can inform on the vulnerability of each life stage to thermal stress a result of temperature rises, and ultimately communities, to abrupt environmental changes (Seebacher and Franklin, 2012; Small *et al.*, 2015).

Despite a critical need to understand how larvae in particular respond to the environment, most literature emphasizes on the relationships between physical changes and major life history events, such as development, growth and survival, and less so on the stage-specific metabolic responses (Paschke *et al.*, 2010; Schiffer *et al.*, 2014; Alter *et al.*, 2015; Small *et al.*, 2015; Leiva *et al.*, 2018). Larval responses to environmental change of sesarmid and other mangrove-associated crabs have been addressed through respirometric research on embryos, the physiological responses to salinity stress and their stage-specific osmoregulatory capabilities (Anger and Charmantier, 2000; Charmantier *et al.*, 2002; Diele and Simith, 2006; Simoni *et al.*, 2013; Simith *et al.*, 2014). The understanding of how mangrove crab larvae respond to acute and possibly transient temperatures is, however, still lacking to ascertain how persistent climate anomalies will influence the larval supply and recruitment success to maintain functional mangrove crab populations.

The present study hence aimed to investigate the effects of acute temperature changes within the thermal range experienced during austral summer, the reproductive season of brachyurans on the east coast of South Africa (Papadopoulos *et al.* 2002). Furthermore, the physiological performance consequent to abrupt thermal changes of stage-specific (zoeal and megalopal) mangrove-associated brachyuran larvae collected at different microhabitats at two South African mangrove sites was investigated. We specifically tested the hypothesis that the fine-scale, recent environmental history of an organism could amplify the interspecific physiological responses of crab larvae by controlling for different microhabitats within the same estuary. Additionally, we hypothesized that larvae subjected to abrupt thermal exposure should not show signs of declining metabolic performance even at the maximum experimental temperature, as the temperatures tested are within the range naturally experienced.

## Materials and methods

### Sampling areas and environmental conditions

**Figure 1:**
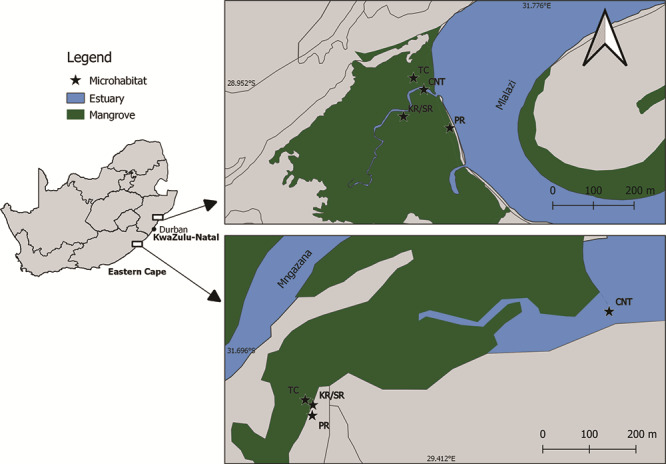
Map of study sites (top, Mlalazi; bottom, Mngazana) on the east coast of South Africa. Microhabitats sampled, knee roots (KRs) or stilt roots (SR), pneumatophores (PRs), tidal creeks (TCs) and controls (CNTs) at each mangrove forest are indicated with a star symbol (★).

Two mangrove forests, the subtropical Mlalazi and warm temperate Mngazana, on the east coast of South Africa were selected for larval collection (Fig. 1). The Mlalazi mangrove forest is estimated to be ~40 ha, dominated by *Bruguiera gymnorhiza*, *Avicennia marina* and a small population of *Rhizophora mucronata* (Peer *et al.*, 2018)*.* The Mngazana mangrove forest is dominated by *A. marina*, followed by *B. gymnorhiza* and the largest stand of *R. mucronata* in South Africa (Peer *et al.*, 2018).

Microhabitats are defined as localized, fine-scale environments encompassing the minimum habitable unit (<2 m) of different structural zones [complexity of root systems, tidal creeks (TCs) and open water] pertaining to life history stage and scale of organisms targeted (Morris, 1987; Smith and Hindell, 2005). Microhabitats identified from both mangrove forests were identified and ranked arbitrarily from most to least qualitatively complex according to their structural arrangement as follows: the knee roots or stilt roots (KRs/SRs) of *B. gymnorhiza* and *R. mucronata* at Mlalazi and Mngazana, respectively; pneumatophores (PRs) of *A. marina*; and the soft-bottomed permanently inundated tributary TCs (<1 km in length, <2 m in width) that flow through the mangrove forest. Furthermore, the control (CNT) habitats, situated at the mouth of the estuary at Mngazana and the main inlet into the mangrove forest from the Mlalazi Estuary, respectively, were also chosen (Fig. 1). At Mngazana, due to their low abundance, the KRs of *B. gymnorhiza* were replaced with the SRs of *R. mucronata*. For the purpose of this study, *B. gymnorhiza* and *R. mucronata* were pooled together for consistency and ease of comparison to KRs/SRs.

To assess the monthly variability of the main environmental parameters among microhabitats, temperature and salinity were monitored *in situ* over 2 days from September 2017 to March 2018 (5 trips were carried out within this period) in each experimental microhabitat. Temperature was logged in 5-minute intervals using iButton loggers (Maxim Integrated Products, ColdChain Thermodynamics), placed within 30 cm of each light trap to characterize the environment over the time of the trap deployment. Salinity of the surface water within microhabitats was measured using a handheld sea water refractometer (RedSea) upon the retrieval of each light trap at sunrise. A separate 24-hour monitoring of water temperature was conducted in the TCs in February 2018 to establish the average, minimum and maximum temperatures experienced at the known peak of the sampled brachyuran reproductive season (February) (Papadopoulos *et al.*, 2002).

### Animal collection

**Figure 2:**
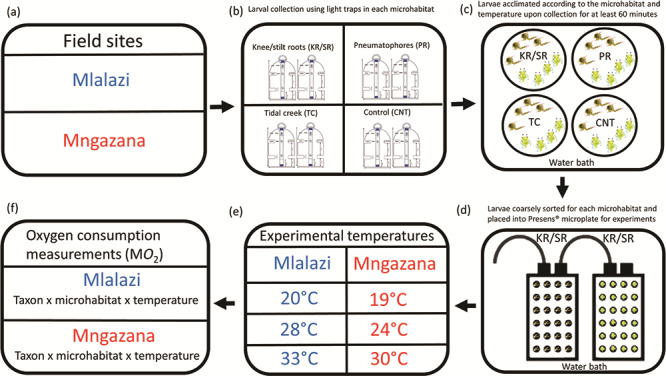
Summary of the experimental design indicating (a) two field sites selected for larval collection; (b) larvae were collected using replicate light traps in each microhabitat during each sampling night (4x2); (c) larvae were acclimated at the on-site laboratory according to habitat sampled, before experiments commenced; (d) larvae from each microhabitat were coarsely sorted into zoeae and megalopae and placed in microplates for ensuing experimental trials; (e) experiments were then carried out according to predetermined temperatures at each study site; and (f) oxygen measurements were recorded from trials for each taxon (after positive identification) at each microhabitat and temperature.

Larvae were collected overnight at each study site using small modified light traps (as Chan et al*.*, 2016), with a spatial resolution of 1.5 m that ensures larvae collected are present in the microhabitats sampled to assess their thermal physiology at three experimental temperatures (Fig. 2). Four areas were identified for each microhabitat type (to maximize and spread larval collection) per study site, where two replicate traps were placed within each microhabitat and at least 5 m from other identified microhabitats to avoid sample overlap within each area, on each sampling night (4 × 2) (Fisher and Bellwood, 2002). The light traps were deployed at each microhabitat for ~12 hours from sunset and retrieved the following day at dawn, around new moon spring tides in February, March and October 2018. The collected samples were transported to an on-site laboratory <5 minutes from the sampling areas. On arrival at the on-site laboratory, each sample was sifted through a 65-μm mesh sieve before being placed into separate 500-ml beakers according to the microhabitat sampled and filled with aerated filtered seawater and acclimated in a water bath for at least 1 hour at the temperature they were collected. After acclimation at the collection temperatures, larval samples were coarsely sorted into zoea or megalopa under a dissecting microscope to avoid additional stress from manipulation before being placed back into the water bath. The temperature was then ramped up or down by 1°C every 15 minutes until the desired experimental temperature was reached to avoid immediate acute heat shock to experimental animals, and for experiments to be completed within 8 hours to minimize bias related to endogenous circadian rhythms (Kelley *et al.*, 2011). Larvae were then allowed to settle at the experimental temperature for at least an additional hour.

### Experimental setup

Three experimental temperatures were selected based on the monitoring period detailed above. With the additional 24-hour *in situ* monitoring period in February 2018, the nominal low, average and high temperatures for each study site were 20°C, 28°C and 33°C, respectively, for Mlalazi and 19°C, 24°C and 30°C, respectively, for Mngazana and subsequently the selected experimental temperature at which oxygen consumption of larvae were measured (Fig. S1). The temperatures selected were typically experienced by larvae during the reproductive season (summer).

The oxygen consumption (*M*O_2_) rates of brachyuran larvae sampled from specific microhabitats were measured separately using an optical fluorescence-based oxygen meter (Sensor dish reader SDR2, PreSens, Germany). A static respirometry system was utilized where up to a maximum of three larvae, depending on their size and life stage (three zoeae or one megalopa), were placed into sealed 80-μL (zoea) or 200-μL (megalopa) wells within a Loligo Systems (Denmark) 24-well glass microplate. Larvae were unfed and kept in the dark during the respirometry trials approximating the results to standard metabolic rate (Clarke, 2004). Additionally, up to four wells on each plate during each trial were filled with filtered estuarine water to control for background respiration. Measurements of oxygen consumption were taken every minute throughout the experimental run, for ~60 minutes, and recorded using the SDR version 4.0.0 software (PreSens, Germany). Oxygen consumption was recorded as the linear change of oxygen content over time per individual well and only the first 30% *p*O_2_ linear decrease in air saturation was used to calculate the oxygen consumption rate as to minimize the effect of stress and hypoxia on oxygen consumption. Lastly, *M*O_2_ was corrected for background respiration and expressed as nmol O_2_ min^−1^ ind^−1^.

At the end of each experimental trial, larvae were preserved separately for photographs to be taken, identification to the lowest possible taxonomic unit and further measurements using a high-powered stereo microscope (Olympus SZX2-ILLB). On limited occasions, if more than one taxon was identified within a single well after the trial was concluded, that replicate was discarded. Dominant taxa identified at both sites included the sesarmid, *Pinnotheres* sp., *Pinnixa* sp. and *Panopeus africanus* stage I zoea and *Neosarmatium africanum*, *Parasesarma catenatum*, *Pinnotheres* sp. and *Metopograpsus thukuhar* megalopa (Table 1). The biovolumes of individuals were estimated using geometric shapes to correct for the volume of each experimental well and calculate individual oxygen consumption. The biovolume of zoeae was estimated from the volume (*V*) of a sphere, ($V=\frac{4}{3}\pi {r}^3$), where *r* represents the radius measured individually. The biovolume of megalopae was estimated from the volume (*V)* of a rectangular prism, (V = l*w*h), where *l* represents the maximum length, *w* the maximum width and *h* the maximum height of each individual (Hillebrand et al., 1999).

**Table 1:** Number of replicate units per taxon, microhabitat and site used for each temperature treatment

Species	Temperature (°C)	CNT	KR/SR	PR	TC
Mlalazi					
	20	25	11	-	-
Sesarmid zoea	28	6	19	-	-
	33	-	6	-	6
	20	4	15	17	12
*Pinnotheres sp.* zoea	28	4	4	38	-
	33	-	14	-	-
*Pinnixa* sp. zoea	33	-	-	59	6
	20	8	30	22	-
*N. africanum* megalopa	28	3	-	8	38
	33	32	35	3	70
	20	8	13	28	16
*P. catenatum* megalopa	28	23	29	15	16
	33	14	15	11	4
	20	10	3	-	6
*Pinnotheres* sp. megalopa	28	11	-	3	-
	33	9	4	-	-
Mngazana					
	19	30	45	33	35
Sesarmid zoea	24	50	10	17	16
	30	28	-	21	27
*Pinnotheres* sp. zoea	24	8	16	-	-
	30	-	-	4	16
*P. africanus* zoea	30	-	4	4	12
*P. catenatum* megalopa	30	-	18	10	10
	19	-	5	4	20
*Pinnotheres* sp. megalopa	24	32	3	6	18
	30	37	37	14	11
*M. thukuhar* megalopa	19	-	12	27	-
	24	-	19	5	4

### Statistical analysis

Temperature and salinity were tested for normality and homogeneity of variances using Shapiro–Wilk and Levene’s test, respectively. These tests showed a violation of the assumptions of normality and homoscedasticity for temperature and salinity at both sites. Temperature and salinity data were therefore compared among microhabitats within each month sampled using Kruskal–Wallis tests. An Aligned Rank Transformation was hence applied on the data to conduct nonparametric factorial analyses of variance among months and microhabitats using the *ARTool* package (Wobbrock *et al.*, 2011). Additionally, to assess the variability of temperature and salinity, the R package *cvequality* (Marwick and Krishnamoorty, 2019) was used to test for significant differences in the coefficient of variation (C_v_) among microhabitats within months using the modified signed-likelihood ratio test (M-SLRT) (Krishnamoorthy and Lee, 2014).

Due to variable numbers of individuals tested for each taxon per microhabitat and temperature (Table 1), separate GLM’s were conducted for each taxon per site, to test for differences among microhabitats at each temperature. Assumptions of normality and homogeneity of residuals were tested using Shapiro–Wilk and Levene’s test, respectively. Where residuals did not meet these assumptions, generalized linear models (GLMs; gamma distribution with a log-link function) were used. Separate GLMs were conducted to test for differences in *M*O_2_ among temperature within each taxon and microhabitat. Only measurements where taxa had sufficient technical replicates from at least two experimental temperatures from each specific microhabitat were included in the analysis (Table S2). Linear regressions were then calculated from an Arrhenius plot on the respiration rates using the equation (Arrhenius, 1889):

(1) \begin{equation*} \ln R=1\text{n}\ a-\frac{E}{k}\ast\frac{1}{T}, \end{equation*}

where *R* is the respiration rate, *a* is taxon specific the normalization constant, *E* is the activation energy (calculated using: *a* = − *E*/*k*), *k* is Boltzmann’s constant and *T* is water temperature in Kelvin.

An ANCOVA was used to test for differences in the activation energy (aE) among taxa, where the taxon was a fixed variable and the inverse of absolute temperature (Kt) was the covariate, after the homogeneity of slopes was confirmed by the interaction term (*F*  _(3,797)_ = 1.56, *P* = 0.197). All significant tests (*P* < 0.05) were followed by Tukey post hoc tests using a Benjamini–Hochberg correction (Benjamini and Hochberg, 1995). All statistical analyses were conducted in the R environment for computing statistics (R v3.3.1) (R Core Team, 2018).

## Results

Salinity at Mlalazi ranged from 7.5–35, with a mode of 20, while salinity at Mngazana varied from 20–38, encompassing a mode of 30 (Fig. S2). Water temperature across all microhabitats showed a mean of 24.7 °C with a range of 16.9–34.5 °C for Mlalazi (Fig. S3a), while the mean for Mngazana was 21.3 °C, with a range of 12.2–30.3 °C (Fig. S3b). There was a significantly increased variability in salinity among microhabitats in September (C_v_ = 14.97, *P =* 0.002) at Mlalazi and January (C_v_ = 11.44, *P* = 0.009) at Mngazana (Table S1). Temperature variability differed significantly among microhabitats within September (C_v_ = 11.47, *P =* 0.009) at Mlalazi. Temperature variability in November (C_v_ = 9.94, *P =* 0.019) and February (C_v_ = 16.93, *P <* 0.001) significantly differed among microhabitats at Mngazana (Table S1). On average, across months, the control areas (CNT) at both sites had the least variability in salinity, while the KR/SR had the largest variability (Table S1). For temperature, the least variable microhabitat at both sites was the TCs and the most variable was the control areas (Table S1). Overall, mean water temperature and salinity were not significantly different among microhabitats within months for both sites, Mlalazi and Mngazana (Figs S2 and S3). There were, however, significant differences in temperature and salinities among months at Mlalazi (*F*_3,43_ = 14.48, *P* < 0.001) and Mngazana (*F*_4,63_ = 34.01, *P* < 0.001).

**Figure 3:**
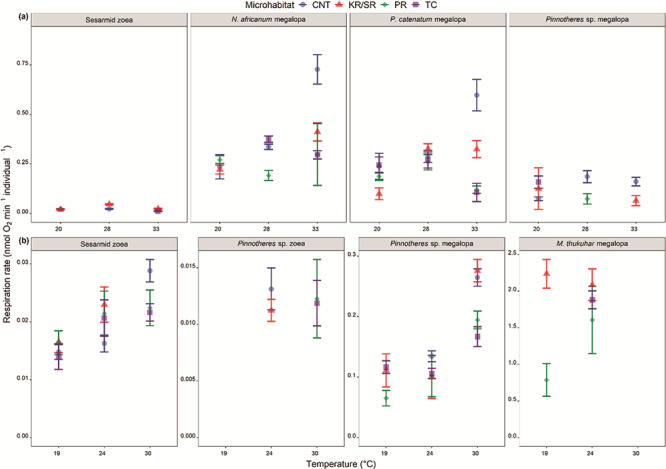
Individual oxygen consumption rates expressed as mean ± SE of dominant brachyuran larvae collected at the CNT (blue circles), KR/SRs (red triangles), PRs (green diamonds) and TCs (purple squares) within the (a) Mlalazi and (b) Mngazana mangrove forest tested at each experimental temperature.

Generally, significant differences in *M*O_2_ within species among microhabitats became apparent only at increased temperatures (Fig. 3). No significant differences in *M*O_2_ were exhibited among larvae sampled from different microhabitats for the majority of taxa at Mlalazi tested at 20 °C (Table 2, Fig. 3). *Parasesarma catenatum* megalopa were the exception (*P =* 0.021, Table 2), with *M*O_2_ significantly higher in specimens collected from the CNT, PR and TC microhabitats than KR/SR (*P* < 0.001, Fig. 3a). At 28 °C, significant differences in *M*O_2_ among microhabitats were observed for *Pinnotheres* sp. zoea (*P =* 0.003, Table 2), *N. africanum* (*P <* 0.001, Table 2) and *Pinnotheres* sp. megalopa (*P =* 0.013, Table 2). *M*O_2_ for individuals collected from the CNT were significantly higher than the PR microhabitat for *N. africanum* (*P* < 0.001) and *Pinnotheres* sp. megalopa (*P* < 0.001, Fig. 3a). At 33 °C, no differences among microhabitats were observed for sesarmid (*P =* 0.757, Table 3) and *Pinnixa* sp. zoea (*P =* 0.791, Table 2). There were, however, differences in *M*O_2_ among microhabitats for *N. africanum* (*P <* 0.001, Table 2), *P. catenatum* (*P <* 0.001, Table 2) and *Pinnotheres* sp. megalopa (*P =* 0.013, Table 2). The *M*O_2_ of specimens from the CNT were again significantly higher than specimens from other microhabitats (Fig. 3a). There were significant differences in *M*O_2_ within species among the three experimental temperatures for sesarmid (*P <* 0.001, Table 4), *N. africanum* (*P <* 0.05, Table 3), *P. catenatum* megalopa (*P <* 0.001, Table 3) and *Pinnotheres* sp. megalopa (*P <* 0.001, Table 3) (Fig. 3a). Sesarmid zoea exhibited significantly higher *M*O_2_ at 28 °C (*P* < 0.05, Fig. 3a) than at the other two experimental temperatures. The *M*O_2_ of all megalopae tested was always highest at 33 °C at the CNT (Fig. 3a, Table 3).

**Table 2:** Outcome of the generalized linear models (GLMs) testing for differences in *M*O_2_ among microhabitats (fixed effect) for each taxon and temperature, at each study site. (χ^2^) chi-squared test statistics, (d.f.) degrees of freedom, (*P*) statistical significance are indicated for each linear model. Significant results are given in bold, different superscript letters for Post hoc tests denote significant differences among microhabitats (*P* < 0.05).

Taxon	Temperature (°C)	χ^2^	d.f.	*P*	Post hoc
Mlalazi					
	20	0.184	1	0.667	
Sesarmid zoea	28	25.868	1	**<0.001**	CNT^a^, KR/SR^b^
	33	20.463	1	**<0.001**	KR/SR^a^, TC^b^
*Pinnotheres sp.* zoea	20	1.903	3	0.592	
	28	4.059	2	0.131	
*Pinnixa* sp. zoea	33	57.676	1	**<0.001**	PR^a^, TC^b^
	20	2.198	2	0.331	
*N. africanum* megalopa	28	25.199	2	**<0.001**	CNT^b^, PR^a^, TC^b^
	33	52.732	3	**<0.001**	CNT^a^, KR/SR^b^, PR^bc^, TC^c^
	20	9.693	3	**0.021**	CNT^ab^, KR/SR^a^, PR^ab^, TC^c^
*P. catenatum* megalopa	28	2.665	3	0.446	
	33	60.792	3	**<0.001**	CNT^c,^KR/SR^b^, PR^a^, TC^a^
	20	4.322	2	0.115	
*Pinnotheres* sp. megalopa	28	5.464	1	**0.019**	CNT^b^, PR^a^
	33	6.868	1	**0.008**	CNT^a^, KR/SR^b^
Mngazana					
Sesarmid zoea	19	1.23	3	0.743	
	24	4.741	3	0.192	
	30	6.611	2	**0.036**	CNT^b^, PR^a^, TC^a^
*Pinnotheres* sp. zoea	24	1.002	1	0.317	
	30	0.007	1	0.929	
*P. africanus* zoea	30	1.479	2	0.477	
*P. catenatum* megalopa	30	8.902	2	**0.202**	KR/SR^a^,PR^b^, TC^ab^
	19	3.907	2	0.141	
*Pinnotheres* sp. megalopa	24	4.849	3	0.183	
	30	22.75	3	**<0.001**	CNT^b^, KR/SR^b^, PR^ab^, TC^a^
*M. thukuhar* megalopa	19	6.393	1	**0.011**	KR/SR^b^, PR^a^
	24	1.156	2	0.561	

**Table 3:** Results from the GLMs testing for differences among temperatures for each taxon, at each study site. (χ^2^) chi-squared test statistic, (d.f.) degrees of freedom and (*P*) statistical significance is indicated for each linear model. Significant results are given in bold, different superscript letters for Post hoc tests denote significant differences among temperatures (*P* < 0.05).

Taxa	Microhabitat	χ^2^	d.f.	*P*	Post hoc
Mlalazi					
Sesarmid zoea	KR/SR	54.657	2	**<0.001**	**20** ^**a**^ **, 28** ^**b**^ **, 33** ^**a**^
*Pinnotheres* sp*.* zoea	KR/SR	1.708	2	0.425	
*N. africanum* megalopa	CNT	12.423	2	**0.002**	**20** ^**a**^ **, 28** ^**b**^ **, 33** ^**c**^
	PR	4.507	2	0.105	
	TC	5.627	1	**0.017**	**28** ^**a**^ **, 33** ^**b**^
*P. catenatum* megalopa	KR/SR	26.972	2	**<0.001**	**20** ^**a**^ **, 28** ^**b**^ **, 33** ^**b**^
	PR	10.912	2	**0.004**	**20** ^**ab**^ **, 28** ^**b**^ **, 33** ^**a**^
	TC	5.419	2	0.066	
	CNT	20.78	2	**<0.001**	**20** ^**a**^ **, 28** ^**a**^ **, 33** ^**b**^
*Pinnotheres* sp. megalopa	CNT	21.46	2	**<0.001**	**20** ^**a**^ **, 28** ^**b**^ **, 33** ^**b**^
Mngazana					
Sesarmid zoea	CNT	39.461	2	**<0.001**	**19** ^**a**^ **, 24** ^**a**^ **, 30** ^**b**^
	PR	3.055	2	0.217	
	TC	7.797	2	**0.041**	**19** ^**a**^ **, 24** ^**ab**^ **, 30** ^**b**^
*Pinnotheres* sp. zoea	CNT;KR/SR;PR;TC	0.001	1	0.964	
*Pinnotheres* sp. megalopa	KR	25.109	2	**<0.001**	**19** ^**a**^ **, 24** ^**a**^ **, 30** ^**b**^
	PR	19.442	2	**<0.001**	**19** ^**a**^ **, 24** ^**a**^ **, 30** ^**b**^
	TC	11.762	2	**0.002**	**19** ^**a**^ **, 24** ^**a**^ **, 30** ^**b**^
*M. thukuhar* megalopa	KR	0.227	1	0.633	
	PR	1.295	1	0.255	

**Table 4:** Parameters of the linear regressions of the Arrhenius plot calculated from the inverse of absolute temperature multiplied by Boltzmann’s constant as a function of the natural logarithm of the respiration rate of each taxon. The (*R*^2^) adjusted R-squared, (*E*) activation energy, (*b*) natural logarithm of the taxon-specific normalization constant and (*P*) significance value are indicated for each linear model. Significant results are given in bold (*P* < 0.05).

Taxon	*R^2^*	*E*	*b*	*P*
Sesarmid zoea	0.09	0.39	11.35	**<0.001**
*Pinnotheres* sp*.* zoea	0.01	0.00	−0.24	0.137
*N. africanum* megalopa	0.27	0.54	18.54	**<0.001**
*P. catenatum* megalopa	0.11	0.58	19.76	**<0.001**
*Pinnotheres* sp. megalopa	0.24	0.68	24.81	**<0.001**
*M. thukuhar* megalopa	0.03	−0.42	−16.26	0.262

At Mngazana, no differences in *M*O_2_ were observed within sesarmid zoea (*P =* 0.176, Table 2) and *Pinnotheres* sp. megalopa (*P =* 0.071, Table 2) at 19 °C; the *M*O_2_ of *M. thukuhar,* however, differed among microhabitats (*P* < 0.001, Table 2), where rates at KR/SR were significantly higher than at PR (*P* < 0.001, Fig. 3b). There were no significant differences in *M*O_2_ among microhabitats within each species at 24 °C (Fig. 3b). At 30 °C, however, differences in *M*O_2_ were found within sesarmid zoea, *Pinnotheres* sp. and *P. catenatum* megalopa (*P <* 0.05, Table 2). For the sesarmid zoea, the *M*O_2_ of specimens collected from the CNT were significantly higher than those from the PR and TC microhabitats (*P* < 0.05, Fig. 3b). The *M*O_2_ of *Pinnotheres* sp. megalopa *M*O_2_ were significantly higher in specimens collected from the CNT and KR/SR microhabitats (*P* < 0.05) than the ones from the TC. The only significant difference in *M*O_2_ among the experimental temperatures within each taxon at Mngazana was exhibited by sesarmid zoea (*P <* 0.001, Table 3) and *Pinnotheres* sp. megalopa (*P <* 0.001, Table 3), where *M*O_2_ were higher at 30 °C when compared to 19 °C and 24 °C.

**Figure 4:**
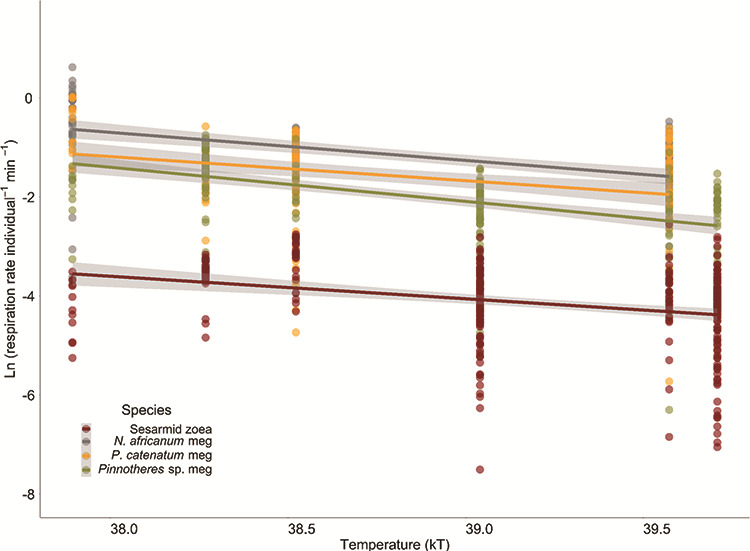
Relationship between temperature and individual respiration rates for specific taxa with significant linear regressions plotted in Arrhenius form. Separate regression lines were fitted for each taxon as the natural logarithm of the metabolic rate as a function of inverse absolute temperature (K) multiplied by the Boltzmann constant (0.0000862 eV K-1). Grey bands indicate the 95% confidence interval of each regression.

The natural log of the respiration rate of *M. thukuhar* megalopa and *Pinnotheres* sp. zoea increased with increasing absolute temperature and thus was not compatible with the Arrhenius equation (Fig. 4, Table 3). Activation energy (eA) extracted from the linearized slopes for the rest of the taxa ranged between 0.39 and 0.68 eV (Table 4). There was a significant difference in eA among all taxa (ANCOVA, *F*  _(3,797)_ = 460.77, *P* < 0.001) and megalopae had higher eAs than sesarmid zoeae (Table S2).

## Discussion

Overall, an increase in temperature resulted in stage-specific responses of brachyuran larvae in *M*O_2_. Invertebrate larvae sampled at specific mangrove microhabitats exhibited differences in their metabolic responses to increasing temperatures, which became more pronounced in the advanced developmental stages. Here, metabolic responses to increased temperature changed according to ontogeny. The zoeae displayed greater thermal sensitivity than megalopa in this study, which could be due to different selective pressures owing to an organism’s environmental history or life history strategy (Dell *et al.*, 2011; Tagliarolo *et al.*, 2018).

The depression in *M*O_2_ at the maximum experimental temperature indicates the energetic limitations of zoeae in the Mlalazi Estuary (Schiffer *et al.*, 2014; Small *et al.*, 2015; Leiva *et al.*, 2018). The metabolic rates of crustaceans living above their thermal optimums have indeed been observed to plateau or sharply decrease (Frederich and Pörtner, 2000; Magozzi and Calosi, 2015). The optimum temperature for an organism’s metabolic rate is usually in the centre of its thermal tolerance range. As such, a decrease in metabolic rates generally occurs when organisms experience temperatures beyond their optimum (pejus temperatures); yet survival is still possible, but becomes deleterious (Frederich and Pörtner, 2000; Pörtner *et al.*, 2000; Pörtner, 2001, 2002).

The thermal sensitivity of zoeae at the temperature extremes within mangrove systems is likely influenced by their early life history strategy. Recently hatched zoeae rely on yolk reserves (Anger, 1995) and employ a rapid seaward export for continued development in neritic waters (Dittel and Epifanio, 1990; Papadopoulos *et al.*, 2002; Paula *et al.*, 2004). This migration from the mangroves to more stable offshore environmental conditions (Paula *et al.*, 2004) suggests that early stage larvae are not evolutionary equipped to cope with acute temperature increases typical of intertidal systems (Miller *et al.*, 2013). The short residency within the mangrove before export is thus dependent on the environmental conditions (temperature) and is of key importance for population persistence if zoeae regularly experience deleteriously high temperatures. In contrast, competent megalopae, after having developed offshore, return back to their settlement areas within mangroves using the nocturnal flood tide (Dittel and Epifanio, 1990; Paula *et al.*, 2004; Ragionieri *et al.*, 2015). Megalopae returning to the intertidal mangroves to settle could be better adapted to acute temperature changes and afford a costly high thermal tolerance through capitalizing energy accumulated during offshore development (Miller *et al.*, 2013).

The megalopae, in this study, generally showed no signs of metabolic failure when exposed to the highest temperatures at the control in Mlalazi and across habitats at Mngazana, confirming that stage-specific changes contribute to the thermal ranges within which mangrove crab larvae survive (Sastry and McCarthy, 1973; Small *et al.*, 2015). An ontogenetic shift in optimum temperature can be attributed to specific energetic demands linked to the development of osmoregulatory, respiratory and cardiovascular structures and functions during a particular life stage (Spicer and Strömberg, 2003; Small *et al.*, 2015). The results in this study with regards to thermal sensitivity suggests that limitations in the aerobic pathway in early stage larvae affect the optimum temperatures ranges in which they are able to grow efficiently and survive to further their ontogenetic development through moulting (Weiss *et al.*, 2012).

At the low range of experimental temperatures, larvae from both study sites showed no difference in *M*O_2_ among microhabitats from where they were sampled, with the exception of *M. thukuhar* at Mngazana and *P. catenatum* at Mlalazi. When the experimental temperature was increased, differences in *M*O_2_ among microhabitats became evident. These differences reaffirm how the effect of an organisms’ environmental history can shape its physiological performance after short-term exposures to increased temperature (Castillo and Helmuth, 2005; Leiva *et al.*, 2016, 2018). Interestingly, temperature and salinity measurements did not differ within each month in which the study was carried out. This postulates that the average temperature and salinity experienced among microhabitats have less of an effect on physiology, but rather additional factors, not taken into account in this study (e.g. water depth and flow, dissolved oxygen, turbidity, prey availability, state of the tide, timing of the moult cycle), could play a role in affecting the metabolic rates of larval brachyurans. The metabolic rates observed in organisms collected at the control could be due to the variability in the thermal conditions exerted upon larvae, since the control was most variable in temperature. This suggests that larvae occurring at the control are subjected to a more diverse range of thermal conditions that could influence their metabolism. Larvae collected from the control microhabitats exhibited higher metabolic rates at increasing temperatures than larvae collected from any other mangrove microhabitat. The constant inundation of water at the control sites compared to the other microhabitat s (except the TC) could be a result of the quality of water (oxygen enriched due to tidal flushing) and increased predation pressure where more energy would have been allocated to elicit a flight-type swimming behaviour (Mitchell *et al.*, 2017; Briceño *et al.*, 2018). Additionally, the increase in *M*O2 of megalopa sampled among microhabitats could be due to the strategies of individuals in different phases in their development as returning megalopa sampled from the control (indicating re-entry into mangrove areas) accumulate energy reserved through constant feeding in its early stages likely reflected in increased metabolism (Anger *et al.*, 1989). Once it reaches its settlement habitat, megalopa converts a large part of its energy reserves to epidermal protein (Anger *et al.*, 1989). Furthermore, the lack of celerity and physical forcing of water within the TCs as compared to the control areas could negatively affect the dissolved oxygen availability, possibly explaining the depressed metabolic rates of larvae from this microhabitat (Knight *et al.*, 2013).

Some logistic limitations to robustly satisfy the experimental design should be acknowledged, owing to the difficulty in collecting sufficient numbers of larvae for each targeted taxon, life stage at the different microhabitats to be tested at the experimental temperatures to allow for a full orthogonal comparison. Furthermore, variability in the localized conditions in water masses (dissolved oxygen, turbulence, food concentrations) and the precise duration of larval exposure to microhabitat conditions was not directly quantified in this study, which partly limits the interpretation of the data. Fine-scale spatial differences of larvae collected at different microhabitats are, however, evident, particularly in late stage larvae for which permanence in a given microhabitat due to recruitment is plausible. The data presented here also represent the first of its kind study on wild-caught, rather than laboratory-reared, brachyuran larvae investigating the links between microhabitats, temperature and oxygen consumption, as a proxy for metabolic rate.

In summary, the ontogenetic patterns observed in the metabolic responses according to the microhabitat from which they originated provides further evidence to how the environmental history (even temporary) of an organism can influence its physiology to short-term acute temperature changes. Microhabitats in aquatic environments may be used to minimize thermal stress, particularly in shallow coastal habitats (Gordon *et al.*, 1985; Heath *et al.*, 1993; Levin, 2003; Curtis and McGaw, 2012; Monaco *et al.*, 2015). If organisms have the ability to exploit these microhabitats, but their availability is limiting, it is expected that some individuals will be excluded, unable to access these refugia to avoid lethal or sublethal heat stress; however, this does not seem to the case for brachyuran larvae (Beck, 1997; Mota *et al.*, 2015; Lima *et al.*, 2016). Efforts to rehabilitate degraded mangals have mainly focused on monospecific planting of *Rhizophora* spp., with little to no success in the regeneration of forests, fauna and functionality of these systems (Lewis *et al.*, 2019). Here, we show that structural diversity of available habitat (Vorsatz *et al.*, 2021) not only affects abundance (Rönnbäck *et al.*, 1999; Granek and Frasier, 2007), but also the physiological performance of larvae within the mangrove microscape. This underpins the nuances in fine-scale processes of microhabitats and the need to focus on the maintenance of hydrological connectivity among microhabitats for effective conservation of mangroves and larvae of their associated brachyuran fauna. Additional research may be required to quantitatively identify how structural complexity gradients and resultant environmental buffering influence the metabolic rates of brachyuran larvae sufficiently mimicking natural conditions in the laboratory at even finer resolved intra-habitat scales.

## Funding

This work was supported by the National Research Foundation [104911] and The Rufford Foundation small grant award [24522-1].

## Supplementary Material

Supplementary_material_R3_LVDEC2020_coab010
