# Supplementary material for: Fine-scale conditions across mangrove microhabitats and larval ontogeny contributes to the thermal physiology of early stage brachyurans (Crustacea: Decapoda)
Source: Conserv Physiol. 2021 Mar 16;9(1):coab010. doi: 10.1093/conphys/coab010 (PMC8059134; doi:10.1093/conphys/coab010)
Supplement: Supplementary_material_R3_LVDEC2020_coab010 [file supplementary_material_r3_lvdec2020_coab010.docx]

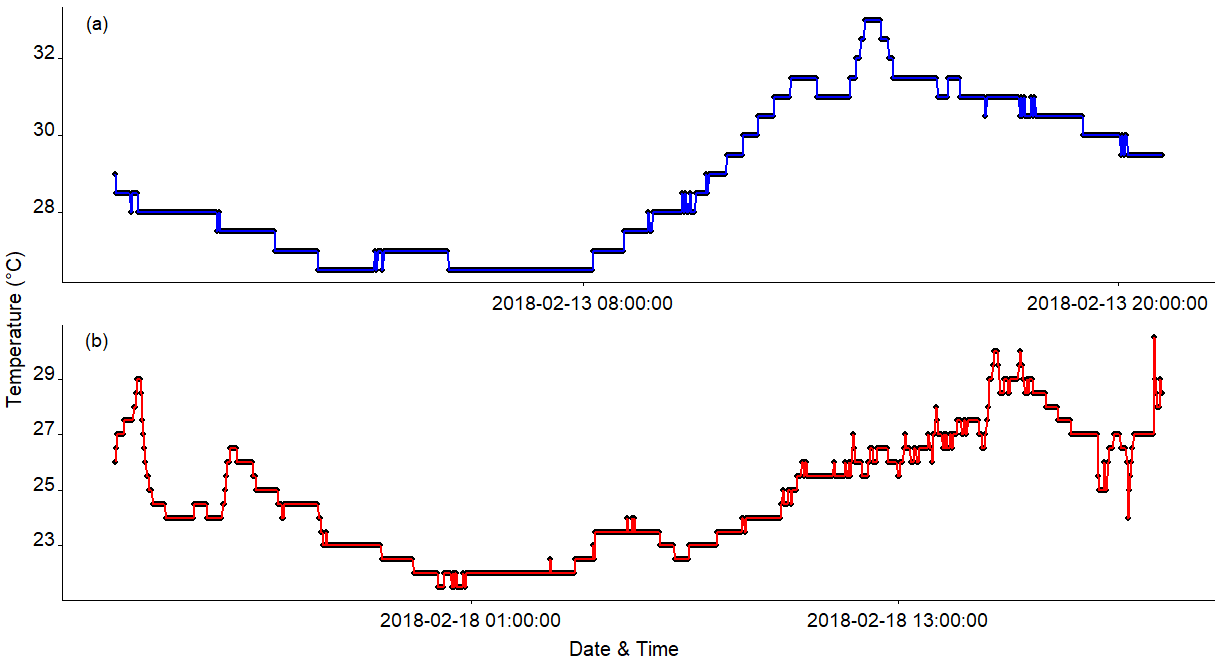


Figure S1. Line graphs of 24-hour temperature monitoring in the tidal creek of A) Mlalazi (blue) and B) Mngazana (red).


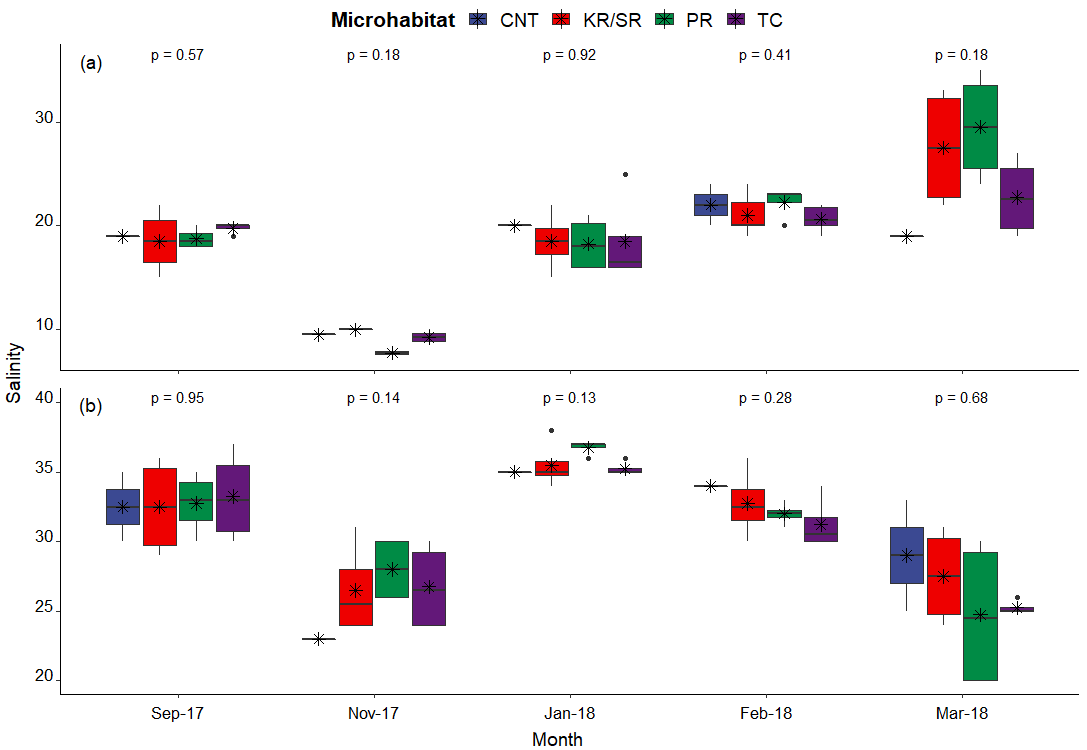


Figure S2. Box plots of salinity at (a) Mlalazi and (b) Mngazana at each microhabitat sampled per month at Mlalazi. The 25 and 75% percentiles are represented by the lower and upper limits of each box; the horizontal line indicates the median, the vertical lines of each box indicate 1.5x above and below the interquartile range, the asterisk (*) indicate the mean and the dark circles (●) show outliers. There were no significant difference in salinity among habitats within each month for both Mlalazi and Mngazana


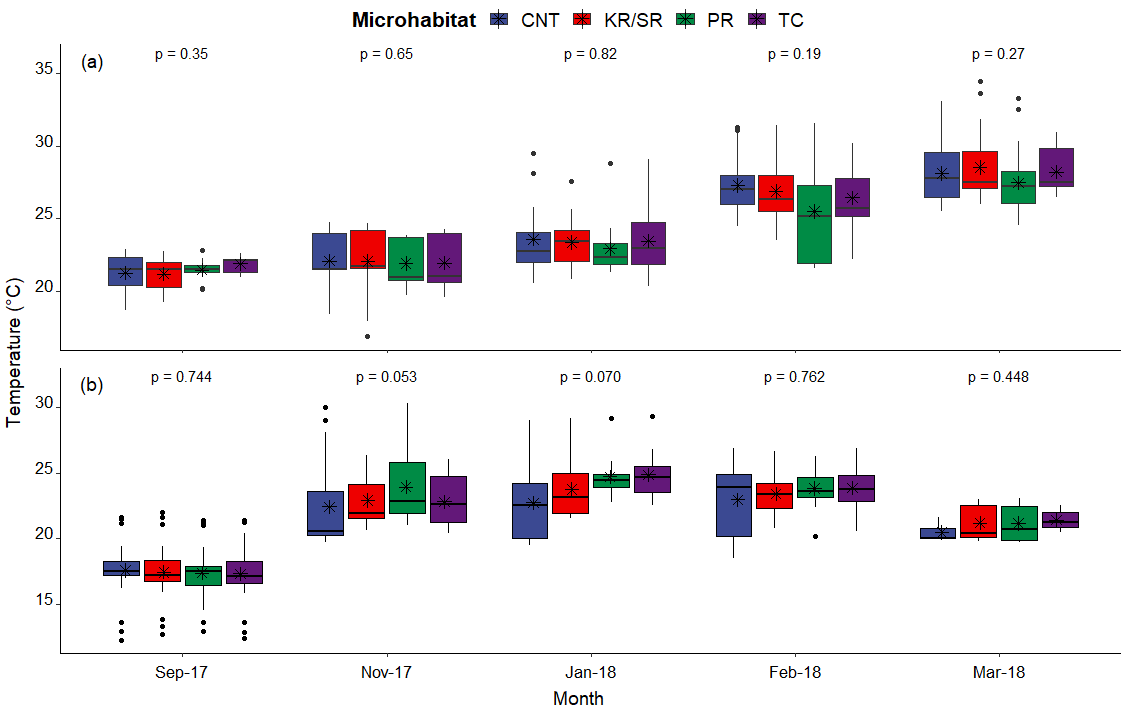


Figure S3. Box plot of temperature at (a) Mlalazi and (b) Mngazana of each microhabitat sampled per month. The 25 and 75% percentiles are represented by the lower and upper limits of each box; the horizontal line indicates the median, the vertical lines of each box indicate 1.5x above and below the interquartile range, the asterisk (*) indicates the mean and the dark circles (●) show outliers. There were no significant difference in temperature among habitats within each month for both Mlalazi and Mngazana

Table S1. Range and variability in salinity and temperature for each microhabitat within each month of environmental characterisation at Mlalazi and Mngazana. (C_v_) Coefficient of variation, (M-SLRT) modified signed-likelihood ratio test statistic. Significant results are given bold.

| Month | Microhabitat | Salinity min/max | Salinity range | C_v_ | M-SLRT statistic (*p*-value) | Temperature min/max (°C) | Temperature range (°C) | C_v_ | M-SLRT statistic (*p*-value) |
| --- | --- | --- | --- | --- | --- | --- | --- | --- | --- |
| **Mlalazi** | | | | | | | | | |
| Sep-17 | CNT | 19/19 | 0 | 0 | **14.97 (0.002)** | 18.7/22.9 | 4.2 | 6.4 | **11.47 (0.009)** |
|  | KR/SR | 15/22 | 7 | 16.8 |  | 19.2/22.7 | 3.5 | 5.1 |  |
|  | PR | 18/20 | 2 | 5.1 |  | 20.1/22.8 | 2.7 | 3.4 |  |
|  | TC | 19/20 | 1 | 2.5 |  | 21/22.6 | 1.6 | 2.5 |  |
| Nov-17 | CNT | 9.5/10.5 | 1 | 7.1 | 4.1 (0.252) | 18.4/24.7 | 6.3 | 9.3 | 2.71 (0.437) |
|  | KR/SR | 10/10 | 0 | 0 |  | 16.9/24.7 | 7.8 | 11.4 |  |
|  | PR | 7.5/8 | 0.5 | 4.6 |  | 19.7/23.9 | 4.2 | 7.3 |  |
|  | TC | 8.5/10 | 1.5 | 11.5 |  | 19./24.2 | 4.7 | 8.2 |  |
| Jan-18 | CNT | 20/21 | 1 | 3.5 | 2.36 (0.51) | 20.5/29.5 | 9 | 11.1 | 1.88 (0.596) |
|  | KR/SR | 15/22 | 7 | 15.6 |  | 20.8/27.6 | 6.7 | 8 |  |
|  | PR | 16/21 | 5 | 14.4 |  | 21.3/28.8 | 7.5 | 8.3 |  |
|  | TC | 16/25 | 9 | 23.6 |  | 20.4/29.1 | 8.7 | 10.3 |  |
| Feb-18 | CNT | 20/24 | 4 | 9.1 | 1.34 (0.726) | 24.5/31.3 | 6.8 | 7.9 | 7.02 (0.071) |
|  | KR/SR | 19/24 | 5 | 9.5 |  | 23.5/31.4 | 7.8 | 8.8 |  |
|  | PR | 20/23 | 3 | 6.7 |  | 21.6/31.5 | 10 | 13.5 |  |
|  | TC | 19/22 | 3 | 5.9 |  | 22.2/30.2 | 8 | 8.2 |  |
| Mar-18 | CNT | 19/20 | 1 | 3.6 | 1.48 (0.682) | 25.5/33.1 | 7.6 | 7.7 | 4.44 (0.217) |
|  | KR/SR | 22/33 | 11 | 21.1 |  | 26/34.5 | 8.4 | 8.7 |  |
|  | PR | 24/35 | 11 | 18 |  | 24.5/33.3 | 8.7 | 8.9 |  |
|  | TC | 19/27 | 8 | 17 |  | 26.5/30.9 | 4.4 | 5.5 |  |
| **Mngazana** | | | | | | | | | |
| Sep-17 | CNT | 30/35 | 5 | 10.9 | 0.66 (0.88) | 12.2/21.6 | 9.4 | 13 | 0.12 (0.988) |
|  | KR/SR | 29/36 | 7 | 10.8 |  | 12.7/22 | 9.3 | 13 |  |
|  | PR | 30/35 | 5 | 6.8 |  | 12.9/21.4 | 8.5 | 12.2 |  |
|  | TC | 30/37 | 7 | 9.9 |  | 12.4/21.4 | 9 | 13 |  |
| Nov-17 | CNT | 23/23 | 0 | 0 | 2.83 (0.417) | 19.7/30 | 10.3 | 15.7 | **9.94 (0.019)** |
|  | KR/SR | 24/24 | 7 | 12.5 |  | 20.6/23.6 | 5.7 | 8.4 |  |
|  | PR | 26/30 | 4 | 8.3 |  | 21/30.3 | 9.3 | 11.8 |  |
|  | TC | 24/30 | 6 | 12 |  | 20.4/26 | 5.6 | 8 |  |
| Jan-18 | CNT | 35/35 | 0 | 0 | **11.44 (0.009)** | 19.5/29 | 9.5 | 13 | 8.83 (0.119) |
|  | KR/SR | 34/38 | 4 | 4.9 |  | 21.5/29.2 | 7.7 | 9.9 |  |
|  | PR | 36/36 | 1 | 1.4 |  | 22.7/29.2 | 6.4 | 6.7 |  |
|  | TC | 35/36 | 1 | 1.4 |  | 22.5/29.3 | 6.8 | 7.6 |  |
| Feb-18 | CNT | 34/34 | 0 | 0 | 5.61 (0.131) | 18.5/26.8 | 8.3 | 12.7 | **16.93 (< 0.001)** |
|  | KR/SR | 30/36 | 6 | 7.6 |  | 20.7/26.6 | 5.9 | 6.1 |  |
|  | PR | 31/33 | 2 | 2.6 |  | 20./26.2 | 6.1 | 5.9 |  |
|  | TC | 30/34 | 4 | 6.1 |  | 20./26.8 | 6.3 | 7.3 |  |
| Mar-18 | CNT | 25/33 | 8 | 19.5 | 7.52 (0.056) | 20/21.6 | 1.6 | 3.1 | 5.48 (0.139) |
|  | KR/SR | 24/31 | 7 | 12.8 |  | 19./23 | 3.2 | 6.7 |  |
|  | PR | 20/30 | 10 | 22.2 |  | 19.7/23.1 | 3.4 | 6.8 |  |
|  | TC | 25/26 | 1 | 2 |  | 20.5/22.5 | 2.1 | 3.6 |  |

Table S2. Pairwise comparisons of significant activation energies (eAs) among taxa. Significant results are given in bold.

| Post-hoc pairwise comparisons | estimate | *SE* | d.f | *t*-value | *p* |
| --- | --- | --- | --- | --- | --- |
| *N. africanum* meg vs *P. catenatum* meg | 0.41 | 0.1035 | 797 | 4.03 | **0.004** |
| *N. africanum* meg vs *Pinnotheres* sp. meg | 0.82 | 0.098 | 797 | 8.34 | **<0.001** |
| *N. africanum* meg vs sesarmid zoea | 2.88 | 0.093 | 797 | 30.86 | **< 0.001** |
| *P.catenatum* meg - *Pinnotheres* sp. meg | 0.41 | 0.09 | 797 | 4.33 | < 0.001 |
| *P.catenatum* meg - sesarmid zoea | 2.46 | 0.09 | 797 | 27.83 | **< 0.001** |
| *Pinnotheres* sp. meg vs sesarmid zoea | 2.06 | 0.08 | 797 | 24.88 | **< 0.001** |
